# Effects of vaginal progesterone and placebo on preterm birth and antenatal outcomes in women with singleton pregnancies and short cervix on ultrasound: a meta-analysis

**DOI:** 10.3389/fmed.2024.1328014

**Published:** 2024-04-05

**Authors:** Limin Peng, Yan Gao, Chengkun Yuan, Hongying Kuang

**Affiliations:** ^1^Department of Obstetrics, The First Affiliated Hospital, Heilongjiang University of Chinese Medicine, Harbin, Heilongjiang, China; ^2^Medical Department of First Clinical Medical College, Heilongjiang University of Chinese Medicine, Harbin, China; ^3^Department of Gynecology, The First Affiliated Hospital, Heilongjiang University of Chinese Medicine, Harbin, China

**Keywords:** vaginal progesterone, placebo, short cervix, singleton pregnancy, preterm birth, adverse pregnancy outcomes

## Abstract

**Background:**

Vaginal progesterone in preterm birth and adverse outcomes caused by cervical insufficiency remains controversial. To address it, the effect of vaginal progesterone on preterm delivery and perinatal outcome of single pregnancy women with short cervix (less than 25 mm) was systematically evaluated by meta-analysis.

**Methods:**

“Vaginal progesterone,” “placebo,” “ultrasound,” “cervix,” “singleton pregnancy,” “preterm birth,” and “antenatal outcomes” were entered to screen clinical studies PubMed, Embase, and the Chinese Biomedical Literature Database (CBM). The study population consisted of women with singleton pregnancies and a short cervix on ultrasound, and were assigned into the progesterone group (*n* = 1,368) and the placebo group (*n* = 1,373). Treatment began after the patient was diagnosed with short cervix until delivery. Neonatal survival rate, Neonatal Intensive Care Unit (NICU) admission rate, respiratory distress syndrome (RDS), intraventricular hemorrhage (IVH), neonatal mortality, and birth weight <1,500 g were analyzed.

**Results:**

A total of 8 articles, totaling 2,741 study subjects, were enrolled. The progesterone group exhibited an obvious reduced rate of preterm birth at <34 weeks (OR = 0.67, 95% CI: 0.53∼0.84; *Z* = 3.53, *P* = 0.004), preterm birth at <32 weeks (OR = 0.46, 95% CI: 0.28∼0.77; *Z* = 2.99, *P* = 0.003), NICU admission rate (OR = 0.45, 95% CI: 0.30∼0.66; Z = 0.15, *P* < 0.0001), RDS rate (OR = 0.42, 95% CI: 0.28∼0.63; *Z* = 4.25, *P* < 0.0001), IVH incidence rate (OR = 0.40, 95% CI: 0.17∼0.95; *Z* = 2.08, *P* = 0.04), neonatal mortality (OR = 0.25, 95% CI: 0.13∼0.46; *Z* = 4.39, *P* < 0.0001), and proportion of neonates with birth weight < 1,500 g (OR = 0.45, 95% CI: 0.32∼0.64; *Z* = 4.50, *P* < 0.0001).

**Conclusion:**

Vaginal progesterone lowered the incidences of preterm birth and adverse pregnancy outcomes in women with singleton pregnancies and a short cervix.

## Background

Preterm birth is a significant pregnancy complication, and according to statistics from the World Health Organization (WHO), nearly 10 million preterm births occur each year, accounting for approximately 10% of all global newborns (1). Research indicates that factors such as male gender, advanced maternal age (35 years and older), ethnic origin, socioeconomic factors, and multiple pregnancies are associated with the occurrence of preterm birth (2). The maternal complications during pregnancy, such as intrauterine infection, bleeding, and hypertension, not only increase the risk of preterm birth (3), but it also presents issues like low birth weight, respiratory distress syndrome, neurological problems, and infection risk for the newborn (4). Cervical insufficiency is considered a major factor contributing to preterm birth (5). Under normal circumstances, the cervix remains closed to keep the fetus secure within the uterus. However, in the case of a short cervix, the cervix may fail to stay effectively closed, making the fetus vulnerable to external pressure and infection, potentially leading to preterm birth or miscarriage (6). A short cervix is also known as cervical incompetence and typically refers to a cervix length of less than 25 mm, although some researchers use 3.0 cm as the critical threshold for a short cervix (7, 8). Due to the cervix opening prematurely, pregnant women are more susceptible to intrauterine infections, which can result in preterm birth or endanger the life of the fetus (6). Some studies suggest that a short cervix may also lead to other antenatal complications, such as premature rupture of membranes, early membrane rupture, or malposition of the fetus and the placenta (9).

Progesterone primary pregnancy promoting hormone, is secreted by the corpus luteum in the ovaries and is crucial in maintaining the integrity of uterine lining during early pregnancy and throughout the course of pregnancy (10). Insufficient progesterone levels can lead to cervical insufficiency, early miscarriage, early pregnancy bleeding, and other pregnancy complications, and can also have an impact on the emotional wellbeing of the mother and the development of the fetus (11). In recent years, vaginal progesterone has been widely used as a therapeutic medication during pregnancy for the treatment or prevention of conditions related to cervical insufficiency and preterm birth. Several studies have indicated that vaginal progesterone can significantly reduce the risk of preterm birth caused by cervical insufficiency, especially in cases where pregnant women have a history of preterm birth (12, 13). However, there is still ongoing debate about the effectiveness of progesterone therapy in preventing and managing preterm birth. Some studies suggest that the effectiveness of progesterone therapy may be limited (14).

To gain a better comprehension of progesterone in prevention and management of preterm birth, more comprehensive research and comprehensive analysis are needed. Therefore, this work employed a meta-analysis approach to comprehensively investigate whether progesterone treatment had a remarkable impact on preventing preterm birth and antenatal outcomes. The aim was to evaluate the effectiveness of progesterone treatment in improving cervical function and lowering the possibility of preterm birth, providing additional evidence for medical decision-making, offering better guidance for the health and antenatal management of pregnant women, and further advancing research in this field.

## Materials and methods

### Research design

This work focused on women with singleton pregnancies who have been diagnosed with a short cervix through ultrasound examination. It employed a meta-analysis approach as the research strategy. Based on the differences in the measures taken for the included study subjects, the participants were assigned into a vaginal progesterone treatment group and a placebo treatment group. The work aimed to compare the differences in preterm birth rates at different gestational weeks and the related antenatal outcomes between these two groups.

### Methods for literature screening

Before conducting a literature search, it is essential to clarify the research question and objectives. During the literature search process, keywords related to the research topic, such as “vaginal progesterone,” “placebo,” “ultrasound,” “cervix,” “singleton pregnancy,” “preterm birth,” and “antenatal outcomes,” should be used. These keywords were adopted to search in various medical literature resources and databases, encompassing PubMed, Embase, Cochrane Library, Springer, Science Direct, China National Knowledge Infrastructure (CNKI), Wanfang Data, VIP, and China Biomedical Literature Database (CBM). In each database, it would search for the following string: “Vaginal Progesterone” AND “Placebo” AND “Ultrasound” AND “Cervix” “Singleton Pregnancy” AND “Preterm Birth” AND “Antenatal Outcomes.” The search timeframe should cover from the inception of the database to July 2023. Once the search results were retrieved, the next steps included preliminary screening to exclude literature that did not meet the inclusion criteria. This preliminary screening helped narrow down the selection to potentially relevant studies. After this, a full-text review of the selected literature was conducted to judge whether they met the inclusion criteria. This step involved a more in-depth examination of each paper, including the sections “Materials and methods, Results, and Discussion.” Additionally, manual searches for the references and related literature of the selected papers should be performed to ensure that no key studies were missed. This comprehensive approach would help gather a robust set of relevant studies for your meta-analysis.

### Methods for enrolling and excluding the articles

The studies enrolled had to satisfy all the following conditions. (1) Study type: clinical studies, such as randomized controlled trials (RCTs), cohort studies, and case-control studies, were included. (2) Participants must be women with a single pregnancy who had undergone ultrasound testing during pregnancy to determine cervical shortening, with a cervix smaller than 25 mm. (3) When the patient was diagnosed with short cervix, the gestational age should be less than 24 weeks, and at least 34 weeks at delivery. (4) Treatment comparison: enrolled studies must have compared the treatment effects of vaginal progesterone with a placebo. (5) Primary outcomes: the enrolled studies must have reported at least the preterm birth rate and key outcome indicators related to antenatal outcomes, such as neonatal survival rate, neonatal admission to the neonatal intensive care unit (NICU), respiratory distress syndrome (RDS), intraventricular hemorrhage (IVH), neonatal mortality, and birth weight <1,500 g. (6) Complete data: the articles must provide sufficient data. (7) Language of literature: the language of the research literature will be restricted to common languages like English and Chinese to ensure understanding and analysis of the studies.

Articles with any of following conditions had to be excluded. (1) Non-clinical studies, such as reviews, editorial opinion articles, case reports, and animal studies, were excluded. (2) Non-singleton pregnancies: participants with multiple pregnancies (e.g., twins and triplets) were excluded. (3) No cervical length measurement: studies that did not perform ultrasound examinations to determine cervical length in participants were excluded. (4) No control group: studies that did not compare the treatment with vaginal progesterone to a control group were excluded. (5) No primary outcome data: studies that did not provide data on preterm birth rates and antenatal outcomes were excluded. (6) Duplicate publications: those that had already been published in other journals as duplicate research were excluded. (7) Language of literature: literature in languages other than English or Chinese was excluded, unless suitable translations were available for use.

### Extraction of required data

Following the screening of literature building upon the above criteria, the following data were collected and extracted:

(1) Record of each study’s title, author(s), publication year, and journal or source.
(2) Study design: specifying the study design type for each study, such as RCTs, cohort studies, or case-control studies.
(3) Sample size and characteristics: this included the number of participants in both the treatment and control groups, their ages, and other relevant characteristics.
(4) Cervical length measurement: details about the range and threshold values for cervical length measurement.
(5) Outcome measures: neonatal survival rate, NICU, RDS, IVH, neonatal mortality, and birth weight <1,500 g, among others.

Quality assessment of studies is a crucial step in meta-analysis, primarily evaluating aspects such as randomization, blinding, and sample size. Tools provided by the Cochrane Collaboration are commonly utilized for a quality assessment on included literature and potential biases, including selection bias, recall bias, and detection bias, among others. For studies that do not meet the criteria, sensitivity analyses can be conducted to examine their impact on the analysis results. For instance, a meta-analysis can be re-conducted after excluding lower-quality studies. Methods like funnel plots are used to check for the potential of publication bias.

### Methods for statistical analysis

Data extracted are subjected to statistical analysis using Excel software to calculate the combined effect size (typically using the risk ratio) and its 95% confidence interval (CI). Heterogeneity analysis was employed to assess differences among various studies. An initial assessment for heterogeneity was conducted using the chi-squared test to determine the significance of heterogeneity (α = 0.05). *P* < 0.05 indicated a remarkable heterogeneity. The *I*^2^ statistic, available in Rev Man 5.3, was adopted for quantitative assessment of heterogeneity. *I*^2^ < 50% suggested low heterogeneity among studies, and a fixed-effects model (FEM) was typically used for the meta-analysis. *I*^2^ > 50% reflected substantial heterogeneity among studies, a random-effects model (REM) was more appropriate. To assess the potential for publication bias, a funnel plot can be created. The analysis results were often presented in the form of a forest plot, where the effect size and confidence intervals for each study are displayed. *Z*-values and *P*-values from the results are extracted to determine whether inter-group differences are statistically significant. Typically, *P* < 0.05 indicated statistically significant inter-group differences.

## Results

### Retrieved results

**FIGURE 1:**
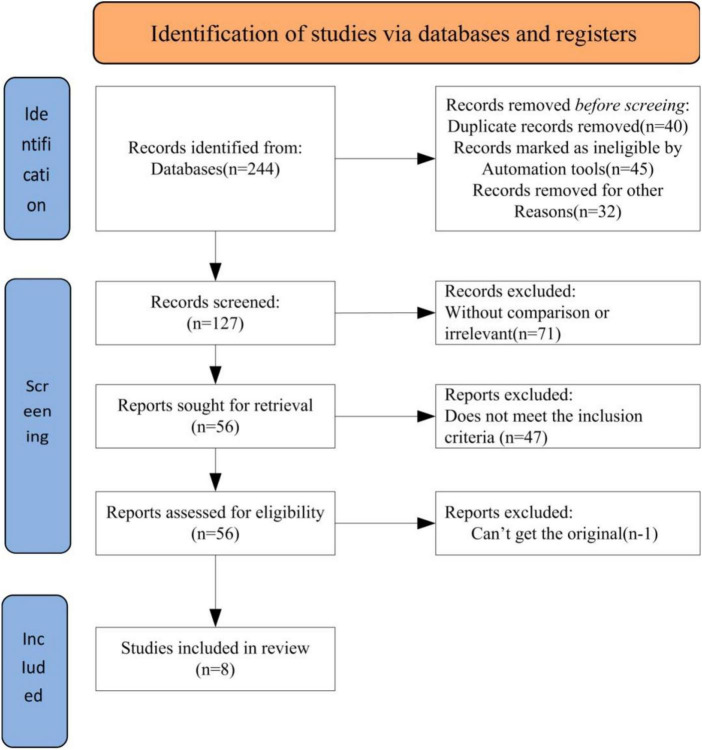
The literature searches and selection process.

The keyword search in online databases yielded a total of 244 relevant articles. After the initial screening, 127 articles were obtained. Further refinement by reviewing article titles and abstracts led to the exclusion of 71 articles that did not align with the research topic, leaving 56 articles. A more in-depth review of the content resulted in the exclusion of 47 articles. After a thorough examination of the remaining 9 articles, one article without access to raw data was excluded. Ultimately, 8 articles were included for analysis. The literature search and selection process were illustrated in Figure 1.

### Brief introduction of enrolled literatures

A total of 8 articles (15–22) were enrolled, comprising a combined population of 2,741 women with ultrasound-diagnosed short cervix in singleton pregnancies. Among them, 1,368 participants were in the progesterone group, and 1,373 were in the placebo group. The basic information was summarized in Table 1.

**TABLE 1:** Brief introduction of these studies.

References	Country	Research type	Total case	Progesterone group	Placebo group
Luxenbourg et al. (15)	United States	Retrospective cohort study	104	48	56
McKay et al. (16)	United States	RCT	468	235	233
Hassan et al. (17)	United States	RCT	468	235	233
Fonseca et al. (18)	United States	RCT	250	125	125
van Os et al. (19)	Holland	RCT	80	41	39
Norman et al. (20)	Britain	RCT	1225	615	610
Azargoon et al. (21)	Iran	RCT	100	50	50
DeFranco et al. (22)	United States	RCT	46	19	27

### Basic characteristics of involved patients

The statistical results for the age, cervical range, cervical length, and treatment methods of the women with ultrasound-diagnosed short cervix in singleton pregnancies from the included studies were presented in Table 2.

**TABLE 2:** Basic characteristics of involved patients.

References	Age (years old)			Cervix length (mm)		Treatment methods		Gestational age (weeks)	
	Progesterone group	Placebo group	Cervix range (mm)	Progesterone group	Placebo group	Progesterone group	Placebo group	Progesterone group	Placebo group
Luxenbourg et al. (15)	30.5 ± 5.5	31.6 ± 5.7	≤25	/	/	Progesterone	No treatment	29.9	30.6
McKay et al. (16)	26.5 ± 5.8	26.2 ± 5.1	10 ∼ 20	17 ± 2.5	17 ± 2.8	Vaginal progesterone gel	Placebo	/	/
Hassan et al. (17)	26.5 ± 5.8	26.2 ± 5.1	10 ∼ 20	17 ± 2.5	17 ± 2.8	Vaginal progesterone gel	Placebo	/	/
Fonseca et al. (18)	29.0 ± 7.4	29.0 ± 7.4	20 ∼ 25	11 ± 3.7	12 ± 3.69	Vaginal progesterone gel	Placebo	23.6	23.4
van Os et al. (19)	31.5 ± 5.6	31.4 ± 5.8	≤30	26 ± 4.44	27 ± 4.50	Vaginal progesterone gel	Placebo	21.7	21.6
Norman et al. (20)	/	/	≤25	28.2 ± 10.6	28.8 ± 11.1	Vaginal progesterone gel	Placebo	/	/
Azargoon et al. (21)	25.4 ± 4.8	24.6 ± 4.9	<28	21.2 ± 5	22.2 ± 4.5	Progesterone	Placebo	/	/
DeFranco et al. (22)	27.4 ± 4.9	25.4 ± 4.8	≤25	244 ± 0.2	22 ± 0.5	Progesterone	Replens	20.4	20.4

### Quality of these studies

**FIGURE 2:**
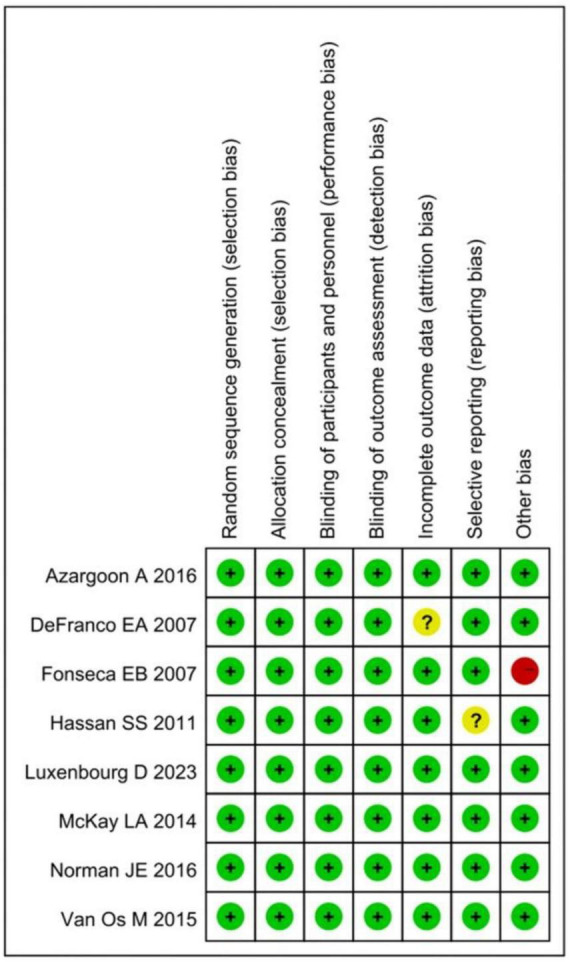
Summary of biased risk assessment in included studies.

Using the Cochrane Reviewer’s Handbook, the quality of the 8 enrolled studies was assessed. A summary chart and bar chart for assessing the risk of bias in the literature were demonstrated in Figure 2. From the figure, it can be observed that all these literatures had “low risk” for random sequence generation (selection bias), allocation concealment (selection bias), blinding of outcome assessment (detection bias), and blinding of participants and personnel (performance bias). One article each had “unclear risk” for incomplete outcome data (attrition bias) and selective reporting (reporting bias), while one article had “high risk” for other bias. Therefore, all the included literature was considered of high quality.

### Impacts of vaginal progesterone on preterm birth of women with ultrasound short cervix and singleton pregnancy

**FIGURE 3:**
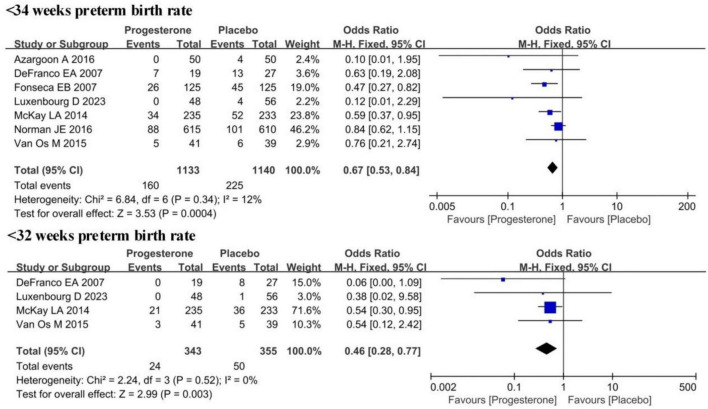
Forest plot of the impact of vaginal progesterone on preterm birth rates in ultrasound-confirmed short cervix singleton pregnancies at <34 weeks and <32 weeks.

Statistical analysis was performed on the preterm birth rates in ultrasound-diagnosed short cervix singleton pregnancy women under both <34 weeks and <32 weeks of gestational age in the progesterone group and the placebo group. No obvious heterogeneity was observed among these studies for both groups (*I*^2^ = 12%, 0%; *P* = 0.34, 0.52). Therefore, a FEM was adopted for the analysis. The results reflected that the preterm birth rate for ultrasound-diagnosed short cervix singleton pregnancy women under <34 weeks of gestational age in the progesterone group was sharply lower in contrast to that in the placebo group (OR = 0.67, 95% CI: 0.53∼0.84; *Z* = 3.53, *P* = 0.004). Similarly, the preterm birth rate for ultrasound-diagnosed short cervix singleton pregnancy women under <32 weeks of gestational age in the progesterone group was lower than that in the placebo group (OR = 0.46, 95% CI: 0.28∼0.77; *Z* = 2.99, *P* = 0.003) (Figure 3).

### The impact of vaginal progesterone on pregnancy outcomes in ultrasound-confirmed short cervix singleton pregnancies

**FIGURE 4:**
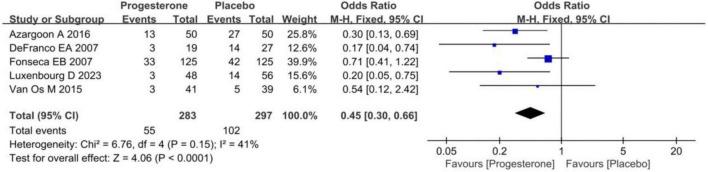
Forest plot of the impact of vaginal progesterone on NICU admission in ultrasound-confirmed short cervix singleton pregnancy after birth.

Statistical analysis was conducted on the proportion of neonatal admissions to the NICU for ultrasound-diagnosed short cervix singleton pregnancy infants in the progesterone group and the placebo group, as demonstrated in Figure 4. No visible heterogeneity was presented (*I*^2^ = 41%; *P* = 0.15). The FEM analysis results implied that the proportion of neonates born to ultrasound-diagnosed short cervix singleton pregnancies and admitted to the NICU was lower in the progesterone group (OR = 0.45, 95% CI: 0.30∼0.66; *Z* = 0.15, *P* < 0.0001).

**FIGURE 5:**
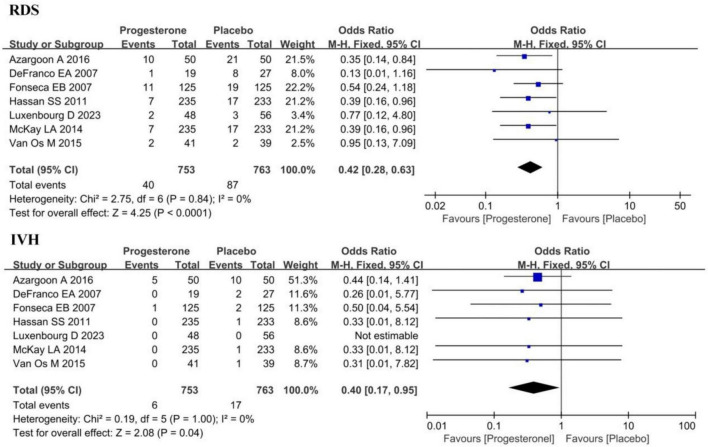
Forest plot of the effect of vaginal progesterone on the incidence of RDS and IVH in ultrasound-confirmed short cervix singleton pregnancy.

Figure 5 illustrated the incidence of RDS and IVH in neonates born to ultrasound-diagnosed short cervix singleton pregnancies in the progesterone group and the placebo group. Heterogeneity was not obvious for both RDS and IVH incidence (*I*^2^ = 0%, 0%; *P* = 0.84, 1.00). Consequently, a FEM was employed here, and its findings revealed that the incidence of RDS in neonates born to ultrasound-diagnosed short cervix singleton pregnancies in the progesterone group was lower (OR = 0.42, 95% CI: 0.28∼0.63; *Z* = 4.25, *P* < 0.0001). Similarly, the incidence of IVH in neonates was also lower in the progesterone group (OR = 0.40, 95% CI: 0.17∼0.95; *Z* = 2.08, *P* = 0.04).

**FIGURE 6:**
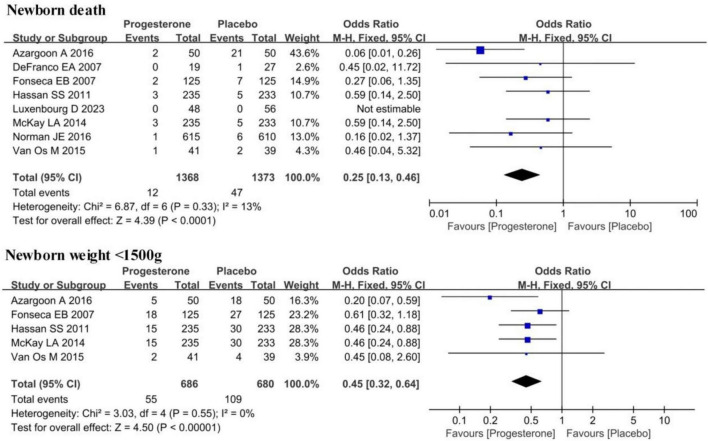
Forest plot for effect of vaginal progesterone on neonatal mortality and the proportion of newborns with birth weight <1,500 g in ultrasound-confirmed short cervix singleton pregnancy.

Figure 6 compared the neonatal mortality rate and the proportion of neonates with a birth weight of <1,500 g among neonates born to ultrasound-diagnosed short cervix singleton pregnancies in the progesterone group and the placebo group. Heterogeneity was not remarkable in these two indicators (*I*^2^ = 13%, 0%; *P* = 0.33, 0.55). The FEM results indicated that the neonatal mortality rate in neonates born to ultrasound-diagnosed short cervix singleton pregnancies in the progesterone group was significantly lower (OR = 0.25, 95% CI: 0.13∼0.46; *Z* = 4.39, *P* < 0.0001). Similarly, the proportion of neonates with a birth weight of <1,500 g was also lower in the progesterone group (OR = 0.45, 95% CI: 0.32∼0.64; *Z* = 4.50, *P* < 0.0001).

### Publication bias

A funnel plot was employed to analyze publication bias in these studies enrolled in this work. The results unveiled that the scatter distribution of the funnel plot for them was symmetrical, indicating no remarkable publication bias.

## Discussion

Preterm birth can lead to a range of health issues, depending on the fetal maturity and the degree of prematurity. Newborns born between 28 and 32 weeks of gestation are at risk of RDS, IVH, and other complications (23, 24). These babies may require care in the NICU to monitor and address potential health problems. For infants born mildly premature between 32 and 34 weeks, their risk is relatively lower, and they typically can breathe independently, with a very high survival rate (25, 26). The results in this work demonstrated that in contrast to the placebo group, patients in the progesterone group experienced a reduced preterm birth rate at both <34 weeks of gestation (OR = 0.67, 95% CI: 0.53∼0.84; *Z* = 3.53, *P* = 0.004) and <32 weeks of gestation (OR = 0.46, 95% CI: 0.28∼0.77; *Z* = 2.99, *P* = 0.003), highlighting the significant reduction in the risk of preterm birth, particularly for births before 34 weeks. This is crucial for extending gestation and improving the maturity of the newborn. The significant reduction in preterm birth before 32 weeks of gestation underscores the effectiveness of progesterone. RDS and IVH are common and critical complications in preterm births. RDS is one of the most common respiratory problems in preterm infants and can impact the normal development of the lungs, especially the maturity of the alveoli and the respiratory tract (27). High IVH is associated with bleeding or hemorrhagic events within the ventricles of the brain, which can lead to damage to brain tissue and have adverse effects on infant neurological development, especially in sensitive brain regions. It may result in cognitive and motor function issues. The study results explicated that progesterone treatment reduces the preterm birth rate, the incidence of RDS and IVH, decreases neonatal mortality, and improves the birth weight of newborns. These are all highly beneficial outcomes for maternal and infant health. Conde-Agudelo et al. (28) evaluated the efficacy of vaginal progesterone in preventing premature delivery and adverse perinatal outcome of twin pregnancy. The results showed that vaginal progesterone could not prevent premature delivery or improve the perinatal outcome of unselected twin pregnancy, but it seemed to reduce the risk of premature delivery in early gestational age and the morbidity and mortality of twins with short cervical ultrasound examination. Romero et al. (29) explored whether vaginal progesterone can prevent premature delivery and improve the perinatal outcome of asymptomatic singleton pregnancy and short-term cervical ultrasound examination in the second trimester. The results showed that vaginal progesterone can reduce the risk of premature delivery and improve the pregnancy outcome of singleton with short-term cervical ultrasound examination in the second trimester, without any obvious harmful effects on children’s neurological development. The results of these meta-analyses are consistent with our research results.

## Conclusion

The results herein revealed that prophylactic vaginal progesterone treatment can reduce preterm birth rates before 34 and 32 weeks of gestation, the incidence of RDS, the incidence of IVH, the proportion of newborns requiring admission to the NICU, neonatal mortality, and the proportion of newborns with a birth weight below 1,500 grams. These findings suggest that progesterone treatment had a greatly positive impact on preterm birth and antenatal outcomes in singleton pregnancies. However, this work was subjected to certain limitations, such as sample size restrictions, differences in study designs, or potential publication bias. Future work should aim to further validate the results of this study. In summary, the results offered valuable insights into intervention strategies for preterm birth and adverse pregnancy outcomes in ultrasound-assessed short cervix singleton pregnancies.

## Author contributions

LP: Writing – original draft, Writing – review and editing. YG: Conceptualization, Software, Writing – original draft, Writing – review and editing. CY: Conceptualization, Investigation, Software, Writing – original draft, Writing – review and editing. HK: Writing – original draft, Writing – review and editing.
